# 115. Variable Use of Diagnostic Codes for Acute Respiratory Infections Across Emergency Departments and Urgent Care Clinics in an Integrated Healthcare System: Implications for Accuracy of Antibiotic Stewardship Metrics

**DOI:** 10.1093/ofid/ofab466.317

**Published:** 2021-12-04

**Authors:** Daniel J Livorsi, Rajeshwari Nair, Michihiko Goto, Eli N Perencevich

**Affiliations:** 1 University of Iowa Carver College of Medicine and Iowa City VA Health Care System, Iowa City, Iowa; 2 The University of Iowa Carver College of Medicine, Iowa City, Iowa; 3 University of Iowa Carver College of Medicine, Iowa City, Iowa; 4 University of Iowa, Iowa City, Iowa

## Abstract

**Background:**

Antibiotic stewardship initiatives can leverage metrics that make peer-peer comparisons. A commonly used metric measures how frequently a clinician prescribes antibiotics for acute respiratory infections (ARIs), as defined by diagnostic codes. However, it is unclear if clinicians differ in their use of ARI diagnostic codes. In this study, we evaluated differences in how frequently clinicians code for ARIs and factors that are associated with the use of ARI diagnostic codes in Emergency Department (ED) and Urgent Care (UC) visits across an integrated healthcare system.

**Methods:**

We analyzed a retrospective cohort of all ED and UC patient-visits across 129 Veterans Affairs medical centers during 2016-2018. ARI visits were identified using ICD-10 codes for acute bronchitis, influenza, pharyngitis, sinusitis, and upper respiratory tract infections for clinicians with 100 or more visits. A generalized linear mixed model with a link logit function that accounted for clustering at the clinician and facility-level was used to calculate median odds ratios (OR) and to identify factors associated with increased likelihood of entering an ARI code.

**Results:**

There were 6,016,499 patient-visits, and 519,389 (8.6%) were coded as an ARI (Table 1). The mean rate of ARI diagnoses across all visits was 8.9% (SD 2.5%) at the facility-level and 7.4% (SD 4.5%) at the clinician-level (Table 2). The median OR was 2.19 (95% CI 2.18, 2.22), suggesting there was between-clinician variation in coding for ARI diagnoses. Visits were significantly more likely to be coded as ARIs if seen by an advanced practice provider (OR=2.36, 95% CI 2.19, 2.54), if a fever was recorded (OR=4.20, 95% CI 4.18, 4.29), and if the visit occurred between December-March (OR=1.97, 95% CI 1.96, 1.98). Approximately 2/5th of the variability (41.4%) in assigning an ARI diagnostic code was explained by differences across individual clinicians.

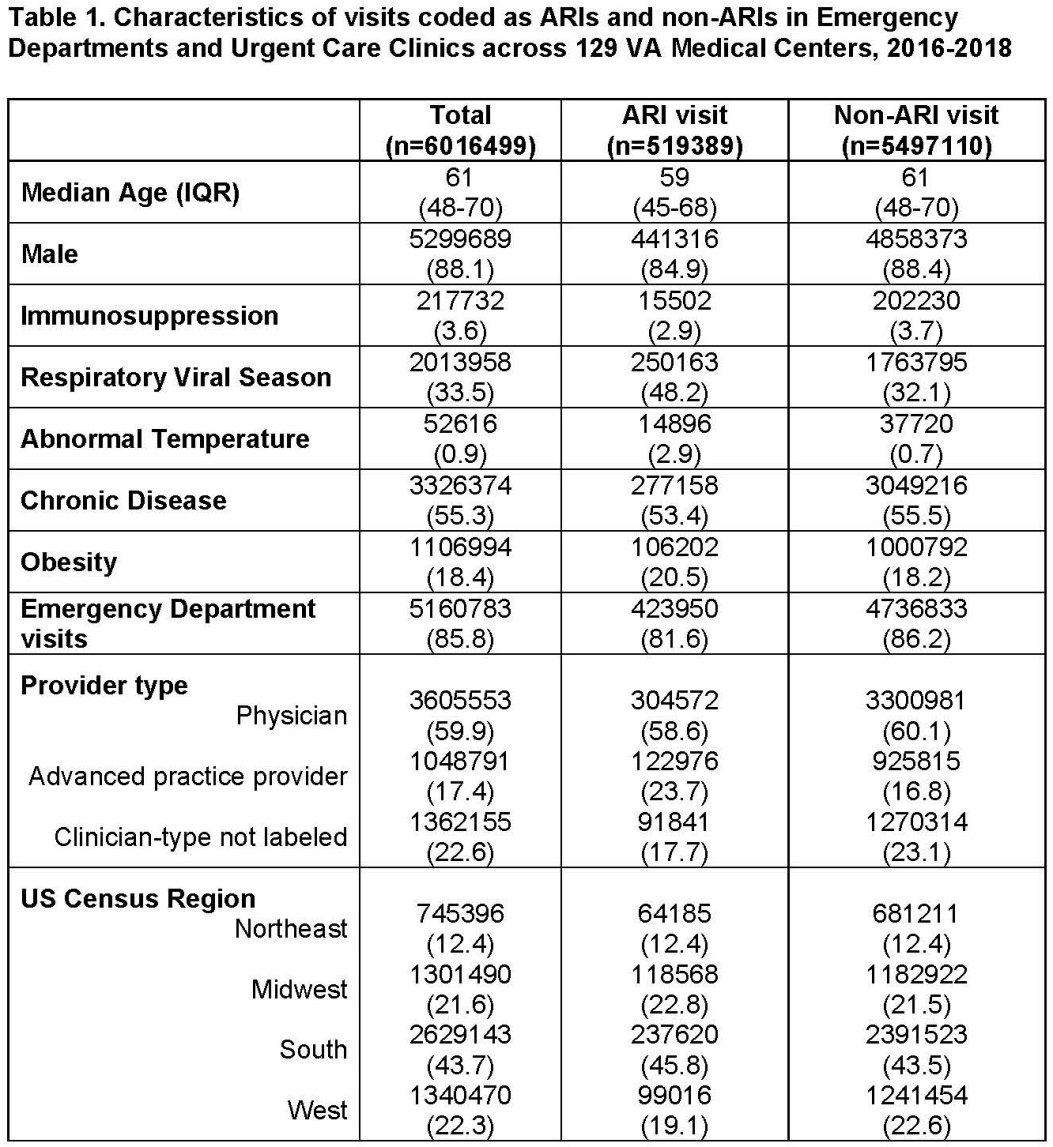

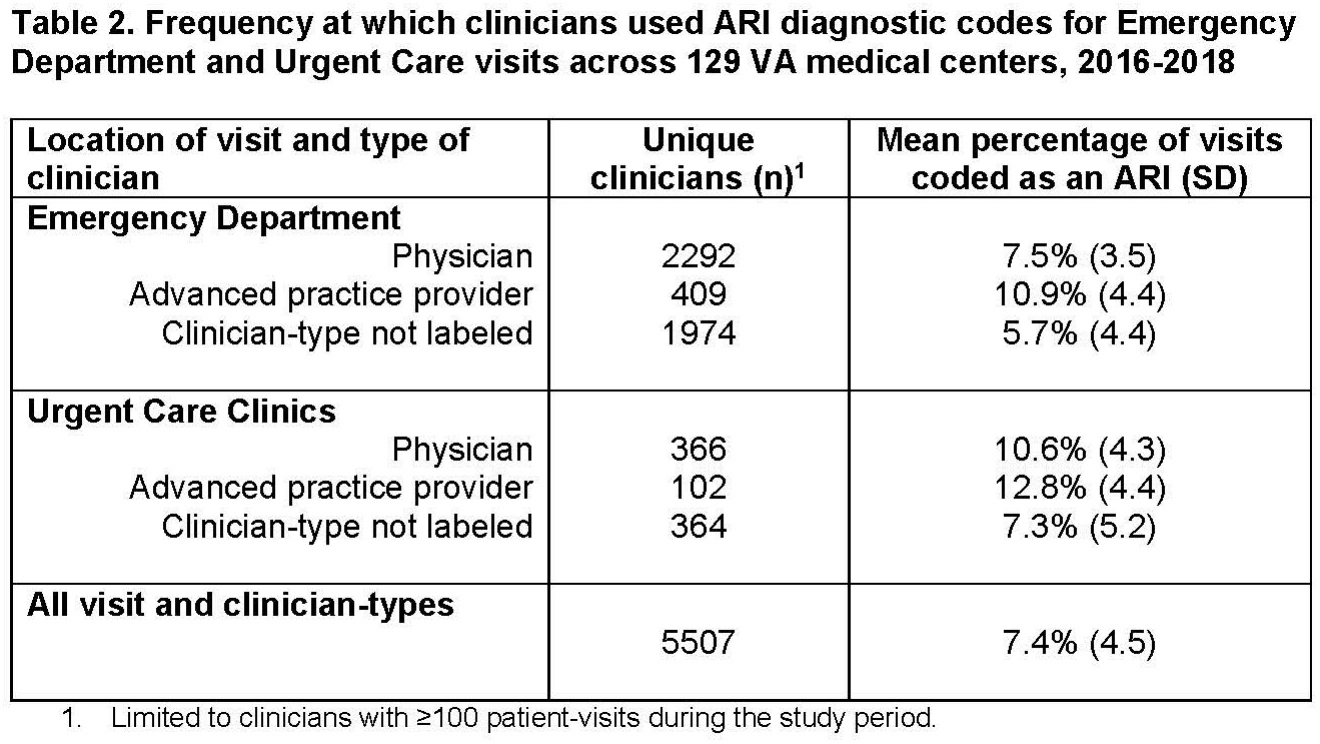

**Conclusion:**

There was substantial variability in how frequently ED and UC clinicians coded a visit as an ARI, and a large proportion of the variability was explained by differences across clinicians. Unmeasured factors could include different approaches to using diagnostic codes. ARI metrics based on diagnostic codes may need to account for differences in clinicians’ coding behavior.

**Disclosures:**

**All Authors**: No reported disclosures

